# Functional Analysis of the Melanin-Associated Gene *CmMR1* in *Coniothyrium minitans*

**DOI:** 10.3389/fmicb.2018.02658

**Published:** 2018-11-08

**Authors:** Chenwei Luo, Huizhang Zhao, Xiaoxiang Yang, Cuicui Qiang, Jiasen Cheng, Jiatao Xie, Tao Chen, Daohong Jiang, Yanping Fu

**Affiliations:** ^1^State Key Laboratory of Agricultural Microbiology, Huazhong Agricultural University, Wuhan, China; ^2^The Provincial Key Lab of Plant Pathology of Hubei Province, College of Plant Science and Technology, Huazhong Agricultural University, Wuhan, China

**Keywords:** *Coniothyrium minitans*, melanin, transcription factor MR, ROS, UV-tolerance

## Abstract

*Coniothyrium minitans* is a sclerotial parasite, which has been investigated for commercial control of crop diseases caused by *Sclerotinia sclerotiorum*. Previously, we obtained a T-DNA insertional mutant, ZS-1TN24363, which did not produce melanin during conidiation. To understand the function of melanin in *C. minitans*, we cloned the gene that was disrupted by the T-DNA insertion, and found that this gene, called *CmMR1*, encoded a putative protein of 1,011 amino acids, which is a homolog of the transcription factor MR. Full-length *CmMR1* contains 3,167 bp, with three exons and two introns. To confirm that the disrupted gene is responsible for the melanin-deficiency of the mutant, *CmMR1* was disrupted and three targeted knockout mutants were obtained. Biological assays showed that the phenotype of the targeted knockout mutants was similar to that of the T-DNA insertional mutant. Furthermore, gene complementation confirmed that *CmMR1* is responsible for the mutant phenotype. *CmMR1* disruption did not affect hyphal growth, conidiation, and parasitization of *C. minitans*, however, the ROS accumulation increased and tolerance to UV light decreased significantly in the mutants. Our result may enhance the understanding of melanin in the ecology of *C. minitans* on molecular level.

## Introduction

*Coniothyrium minitans* is a mycoparasite of *Sclerotinia* spp. and certain species in other related genera (Campbell, 1947; Li et al., 2006), and it parasitizes both the hypha and sclerotia of *Sclerotinia sclerotiorum*. *C. minitans* has been proved to be able to control *Sclerotinia* rots of vegetable crops, stem rot of rapeseed (*Brassica napus*), and head rot of sunflower (Huang, 1981; Whipps and Gerlagh, 1992). *C. minitans* has been developed as a commercial biological agent and been widely used in the United States, European Union, and China. *C. minitans* is a coelomycete, which grows on sclerotia, and produces pycnidia with numerous conidia on or in the sclerotia (Whipps et al., 2008). It also grows well on media such as on potato dextrose agar (PDA) and wheat kernel and forms pycnidia. The pycnidia are initially white, and darken with time. Conidia are dispersed with water droplets from the pycnidia. *C. minitans* synthesizes melanin, which accumulates on the pycnidia, conidia, and the aging hyphae, lending a dark appearance to the entire mature colony.

Many fungi synthesize melanin (Bell and Wheeler, 1986). There are two melanin synthesis pathways; one uses 1,8-dihydroxynaphthalene (DHN) as the initial molecule whereas the other uses L-3,4-dihydroxyphenylalanine (DOPA) (Plonka and Grabacka, 2006). Several studies showed that the melanin synthesis pathways in fungi are regulated by the MR transcription factor. Two transcription factors involved in melanin synthesis were identified (CMR1 and PIG1) in *Colletotrichum lagenarium* and *Magnaporthe grisea*. Upon disruption of *CMR1*, the colony of *C. lagenarium* turned to reddish brown from dark brown. Furthermore, *CMR1* up-regulates the expression of *SCD1* and *THR1*, which encode scytalone dehydratase and T3HN reductase, respectively (Tsuji et al., 2000). Disruption of *CMR1* in *Cochliobolus heterostrophus* leads to melanin shortage, change in colony color to orange or pink, and significant decrease in conidial production. The expression of *SCD1*, *BRN1* (T3HN reductase), and *BRN2* (T4HN reductase) could not be determined in the mutant, whereas the expression of *PKS18* was moderately down-regulated (Eliahu et al., 2007). In *Bipolaris oryzae*, disruption of *BMR1* caused albinism in the mutants, and over-expression of *BMR1* in this fungus enhanced the expression of *PKS*, *SCD1*, and *THR1*. BMR1 levels in the overexpression transformants were ten times that of the wild-type strains (Kihara et al., 2008). Interestingly, disruption of *Amr1* in *Alternaria brassicicola* resulted in impaired accumulation of melanin; however, the pathogenicity was significantly enhanced and many genes encoding hydrolases were up-regulated in the disruption mutants (Cho et al., 2012).

In previous research, we constructed a transfer DNA (T-DNA) insertional library of *C. minitans* strain ZS-1 (Li et al., 2005). A whitish mutant, ZS-1TN24363, was screened from this mutant library. A MR transcription factor gene was disrupted by the T-DNA insertion in ZS-1TN24363. We hypothesized that MR transcription factor regulated melanin in *C. minitans.* In this study, the relationship of the phenotype and gene disruption was confirmed and the potential ecological role of melanin in *C. minitans* was discussed.

## Materials and Methods

### Fungal Strains, Media, and Maintenance

*Coniothyrium minitans* strain ZS-1 was originally isolated from soil at Zhushan county, Hubei province, China; *S. sclerotiorum* strain 1980 is a genome-sequenced strain gifted by Dr. Weidong Chen of the Washington State University. Fungal strains were grown on PDA medium, maintained on PDA slants, and stored at 4°C. Bacteria used for molecular experiments were grown on Luria-Bertani (LB) medium. IM medium and Co-medium were used for fungal transformation mediated by *Agrobacterium tumefaciens* (Gong et al., 2007; Wei et al., 2016; Yang et al., 2016).

### Gene Cloning, RT-PCR Amplification, and Phylogenetic Analysis

Mycelial mass of mutant ZS-1TN24363 was collected from the colonies cultured on PDA medium for DNA extraction using the cetyltrimethylammonium bromide (CTAB) method. HiTAIL-PCR was used to clone the flanking DNA segment of the T-DNA insertional site (Liu and Chen, 2007; Xiao et al., 2014). RNA samples were extracted using a RNA extraction kit of Invitrogen. Complementary DNA (cDNA) was synthesized using a cDNA synthesis kit provided by TransGen Biotech (Beijing, China); DNA sequencing analysis was performed by the TianYiHuiYuan (Guangzhou, China) and the DNA sequence was analyzed using the Basic Local Alignment Search Tool for Nucleotides (BLASTN) against the local database of *C. minitans* to retrieve sequence information of the putative disrupted gene; the expression of the putative disrupted gene was determined using RT-PCR with primer pairs *CmMR1*-F/*CmMR1*-R. *Actin* was checked as a control with Actin-F/Actin-R. The putative disrupted gene was further analyzed using the DNAMAN program to explore the encoded protein, and BLASTP analysis was performed using the National Center of Biotechnology Information’s (NCBI) (GenBank) database. Homologs of other fungi were collected and analyzed, and the phylogenetic tree was constructed using the neighbor-joining method of the MEGA 6.0 program. The expression pattern of melanin synthesis associated gene *CmSCD*, *Cm3HNR* and *Cm4HNR* in strain ZS-1 and the mutant ZS-1TN24363 was monitored by quantitative real time RT-PCR (qRT-PCR) amplification. Data was acquisited and analyzed by using the Bio-Rad CFX Manager^TM^ Software (version 2.0). The relative expression level of each target gene was quantified by the comparative CT method (2^-ΔΔCt^) of quantification. Primer pairs used in this study are listed in Table 1.

**Table 1:** Primers used in this study.

Primer name	Nucleotide sequence (5′ to 3′)	Usage
U-F	ATGTAACGGAATCCCAGGCACTAAC	fragment U
U-R	CGAGGGCAAAGGAATAGAGTAGATGCC GGGGACCACGCAAAACGAAGG	amplification
D-F	GCTCCTTCAATATCATCTTCTGTCGACT CTAGAGATTGGCGTTCAGCGAGCG	fragment D amplification
D-R	CTCGCCACCGCCATCAAGA	
HYG-F	CGGCATCTACTCTATTCCTTTG	fragment HYG
HYG-R	TCTAGAGTCGACAGAAGATGATATT	amplification
YG-F	GGATGCCTCCGCTCGAAGTA	fragment YGD amplification
HY-R	TTGCAAGACCTGCCTGAAACCG	fragment UHY amplification
KOG-F	ATCTGTTCCTACGCTCTTACGCC	confirmation of
KOG-R	CGCTTAGTGAGGGTAGGTGTGCC	knockout and
KOU-F	GAAGAGACGGTGGAGGAGTTTGG	complemented
KOD-R	GCATAGTAGTCACACTGCCTCGTCA	transformants
CO-F	CAGATTTCACGCCGTCTCCAG	construction of
CO-R	GGAAGGGATGGATGAGTGAAGC	complementary vector
NEO-F	CGGTGCCCTGAATGAACTGC	confirmation of
NEO-R	GCGGCGATACCGTAAAGCAC	complemented transformants
*CmMR1*-F	TTGTGGCGAGAGTGGATT	expression of
*CmMR1*-R	CATCTGGCATCGGTGTTC	the disrupted gene
Actin-F	GTCCGTGACATCAAGGAGAAGC	
Actin-R	TTGCCAATGGTGATGACCTGAC	
*CmSCD*-F	GTCGTTCCTCAACAAGTC	expression of
*CmSCD*-R	GATGAAGTGCTGT GTCTT	*CmSCD*
*Cm3HNR*-F	GTTGGTGGCAGGATTATC	expression of
*Cm3HNR*-R	GTTGACAGTG ATCTTCTTCT	*Cm3HNR*
*Cm4HNR*-F	CATCACCGAGGAGAAGTA	expression of
*Cm4HNR*-R	CTTGGATGCGTTGTAGAG	*Cm4HNR*

### Gene Knockout and Complementation

To investigate whether the disrupted gene was responsible for the change in phenotype of the T-DNA insertional mutant ZS-1TN24363, both gene knockout and gene complementation were performed. Split-marker recombination (Zhang et al., 2016) was used to knockout the potential gene. Two genomic DNA segments were amplified using the DNA sample from the wild-type strain ZS-1 as template; one fragment upstream of the target gene (fragment U) was amplified using primer pair U-F/R; the other fragment (fragment D) down-stream of the target gene was amplified using primer pair D-F/R. The hygromycin-resistance gene (HYG) was PCR amplified from the plasmid pKS1004 (Zhang et al., 2016) with primer pairs HYG-F/HYG-R. Fragments U and HYG were mixed as DNA template for PCR amplification with primer pairs U-F and HY-R, and the PCR product was named UHY; fragments D and HYG were mixed as DNA template for PCR amplification with primer pairs YG-F and D-R, and the PCR product was named YGD. The position of the primers was included in Supplementary Figure S3. The PCR products UHY and YGD were mixed and transformed in protoplasts of *C. minitans* wild-type strain ZS-1 using polyethylene glycol (PEG) mediated technique (Wei et al., 2013; Zhang et al., 2016). The transformed protoplasts were spread on regeneration medium containing 50 μg/μL hygromycin and placed in an incubator for 1 week; colonies growing on the medium were transferred to fresh hygromycin-amended PDA plates and further cultured as gene-knockout candidates. Four primers were designed to confirm these candidates; one pair (KOG-F/R) was designed on the target gene, another primer (KOU-F) on the up-stream region of the target gene, and the last primer (KOD-R) on the down-stream region of the target gene. DNA samples were extracted from candidate knockout mutants and amplified by PCR. The primer pairs KOG-F/R, KOU-F/HY-R, YG-F/KOD-R, and HYG-F/HY-R were used to confirm knockout of the target genes.

Based on the local genome database of *C. minitans*, the promoter of the target gene was predicted using the program on http://www.fruitfly.org/seq_tools/promoter.html. The promoter region is about 1,000 nucleotides up-stream of the ATG codon of the target gene. The primer pairs were designed based on the predicted results, and the genomic DNA of strain ZS-1 was used for PCR amplification with primer pairs CO-F/R. Then, the full-length gene with the promoter region was amplified by PCR and digested with *Kpn*I. p3300 was also digested and dephosphorylated. Then PCR products were ligated on the dephosphorylated vector using the T_4_-DNA ligase to obtain a gene complementary vector. This vector was introduced into *A. tumefaciens* strain EHA105 for fungal transformation of the knockout mutants following the method described by Wei et al. (2016) and Yang et al. (2016). The candidate gene-complemented transformants were further confirmed using PCR amplification with primer pairs KOG-F/R, HYG-F/HY-R and NEO-F/R.

### Biological Assay

#### Colony Morphology and Growth Rate Determination

Knockout mutants, complemented tranformants, ZS-1TN24363, and the wild-type strain ZS-1 were activated on PDA plates for 3–4 days. Next, hyphal agar disks were taken from the colony margin with a sterilized hole punch (diameter 5 mm), transferred to the center of fresh 20 mL PDA plates (diameter 90 mm), and incubated at 20°C. The colony diameter was measured every day from the 3rd day to the 10th day. Each strain had three replicates (three plates) and the experiment was repeated thrice.

#### Hyphal Tips Observation

To observe the hyphal tips, all strains mentioned above were activated and transferred on cellophane laid on PDA plates and incubated for 4 days. The colony on cellophane was cut into pieces, and hyphal tips were observed under a light microscope. Typical hyphal tips were imaged with a digital camera (Nikon). This experiment was repeated thrice.

#### Biomass Determination

To determine the biomass of the strains growing on PDA medium, all strains were activated on PDA plates, the hyphal agar disks were punched and placed on cellophane laid on PDA medium, and incubated at 20°C for 10 days. The whole mycelial mass was harvested by rolling the colonies. The weight of fresh mass was measured. Each strain had three replicates, and the experiment was repeated thrice.

#### Conidial Production

To determine the conidial production of the strains, the activated strains were grown on 20 mL PDA plates for 15 days. The whole colony and agar was placed in a mortar with 5 mL distilled water and finely ground, and the liquid was passed through three layers of lens-cleaning paper. The mass was washed again with 5 mL distilled water and the entire liquid was centrifuged at 5,000 rpm for 10 min. The pellet was re-suspended in 1 mL distilled water and the conidial concentration was calculated under the light microscope using a hemocytometer. The conidial production of the entire colony was then calculated. Three colonies of each strain were examined and the experiment was repeated thrice.

#### Parasitic Ability Assay

Two methods were used to determine the parasitic ability of the strains. One was dual culture with strain 1980 of *S. sclerotiorum* according to the method described by Zeng et al. (2014). Mutant or the wild-type strains were placed on one side of the PDA plate (diameter 90 mm) and strain 1980 on the opposite side of the same plate at a distance of 60 mm from the mutant or the wild-type strain. The dual culture was incubated for 20 days. The inhibition zone between the colony of *C. minitans* and that of *S. sclerotiorum* was determined, if any, and the morphologies of the two colonies were also observed. To quantify the parasitic ability of the mutants, *S. sclerotiorum* colony was divided into four iso-distance regions, and five disks from the same region were punched out and placed on fresh PDA plates. After 7 days of incubation, the emerging colonies were judged to be those of *C. minitans* or *S. sclerotiorum*.

Parasitism to sclerotia were further evaluated according to Jiang et al. (2000). *S. sclerotiorum* was inoculated on carrot rods in 250 mL flasks for 30 days to produce sclerotia. Sclerotia were harvested and dried at 37°C for 3 days, and sclerotia with similar sizes were selected to test the parasitic ability of the mutants. Conidia of *C. minitans* were collected from colonies growing on PDA plate for 25 days and diluted with sterilized double distilled water to obtain 10^6^ spores/mL. Sclerotia were submerged in 50% bleach thrice for 5 min to remove the microorganisms on the surface in 70% ethanol for additional 5 min (which was repeated once more), and then washed with double distilled water twice. The surface-sterilized sclerotia were divided into 9 groups with 30 sclerotia per group, and then submerged into the conidial suspension of the wild-type strain ZS-1, mutant ZS-1TN24363, knockout mutants, and complemented transformants for 30 min. Next, the inoculated sclerotia were half-buried in sterilized wet-sand in plates. The plates were sealed and incubated for 30 days. The sclerotia were taken out from the sand and dissected using a surgical knife to rank the rot degree of sclerotia using a method described by Jiang et al. (2000). Each treatment was replicated thrice; desterilized double distilled water was used as the control. The experiments were repeated thrice.

### NBT Staining Test

To determine the reactive oxygen species (ROS) content in the mutant colony, the mutants and the wild-type strain of *C. minitans* were activated and placed on fresh PDA plates for 3 days. Then, mycelia were incubated in 0.05 M phosphate buffer containing 0.05% NBT for 1 h and the reaction was stopped using water-free ethanol. The colonies were observed with naked eyes. Each treatment had three replicates and the experiment was repeated thrice.

### Detection of Tolerance to Ultraviolet (UV) Light

To determine if the mutant conidia could tolerate UV light, conidia were harvested from 10-day-old colonies of mutants and the wild-type strain and diluted with sterilized double distilled water to obtain 10^5^ spores/mL. Spore suspension (100 μL) was carefully spread on PDA plates and dried, and irradiated under UV light (20 J/cm^2^) for 0, 3, 6, and 9 min. The plates were incubated for 36 h, and the germinating conidia were counted under a light microscope. Each strain and mutant had three replicates and this experiment was repeated thrice.

### Statistical Analysis

The one way analysis of variance (ANOVA) of the DPS software was used to analyze the significance of the differences between treatments. Means were separated using the Student Newman–Keuls multiple range test when ANOVA was significant at *P* < 0.01.

## Results

### Biological Characteristics of the T-DNA Insertional Mutant ZS-1TN24363 of *C. minitans*

**FIGURE 1:**
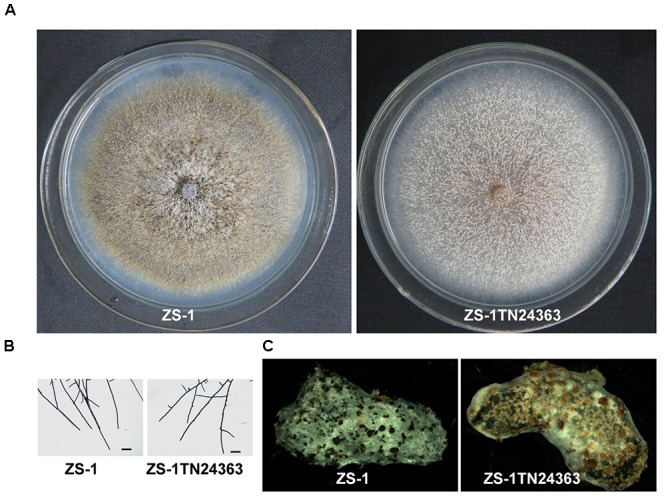
Morphology and parasitization of strain ZS-1 and mutant ZS-1TN24363. **(A)** Colony morphology (PDA, 20°C, 20 days). **(B)** Hyphal tips (PDA, 20°C, 4 days). **(C)** Pycnidia formed on sclerotia of *S. sclerotiorum* (20°C for 30 days). Scale bar means 50 μm.

Similar to the wild-type strain, mutant ZS-1TN24363 could grow on PDA medium (Figure 1A). In addition, the hyphal tips of the mutant were similar to those of the wild-type strain (Figure 1B). The mutant could also form pycnidia and conidia like the wild-type. However, the pycnidia formed were whitish to orange in color, which was significantly different from that of the wild-type strain (dark pycnidia) (Figure 1A), suggesting that this mutant could not synthesize melanin. Further investigation revealed that this mutant could parasitize sclerotia and formed white pycnidia on sclerotia, suggesting that this mutant retained parasitic ability (Figure 1C).

### The T-DNA Disrupted *CmMR1* of *C. minitans*

The flanking 1,000 bp genomic DNA of the T-DNA insertion site was obtained by hiTAIL-PCR (Supplementary Figures S1A,B). This DNA fragment was sequenced and the T-DNA border was identified. The sequence of the flanking DNA was used for searching in a local genome database of *C. minitans* strain ZS-1. We found that the T-DNA insertion possibly disrupted a gene of 3,167 bp. This gene contained three exons and two introns, and encoded a 1,011 amino acid-long protein (Supplementary Figure S1C). BLASTP analysis on the NCBI database revealed that this protein contained three conserved domains, namely a Zn2Cys6 binuclear cluster domain, a Med3 domain that regulates the transcriptional activity of RNA polymerase II, and a fungal-TF-MHR domain that is generally present in a large family of fungal zinc cluster transcription factors (Supplementary Figure S1D). Thus, we presumed that this protein was a transcription factor that belonged to the MHR superfamily. The proteins of MHR superfamily are conserved in fungi. Phylogenetic analysis revealed that this protein was closely related to CMR1, BMR1, and AMR1 (Supplementary Figure S2), and we named this protein CmMR1 (GenBank Acc. No. MF166860).

**FIGURE 2:**
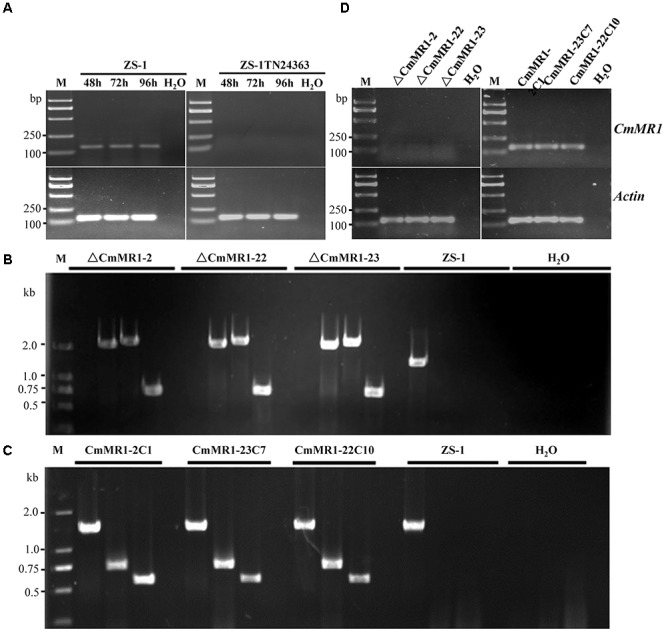
Targeted knockout and complementary of *CmMR1* in *C. minitans.* **(A)** RT-PCR analysis, showing the expression pattern of *CmMR1* from 48 hour post inoculation (hpi) to 96 hpi in strain ZS-1 and in the mutant ZS-1TN24363 with primer pairs *CmMR1*-F/*CmMR1*-R. *Actin* was checked as a control with Actin-F/Actin-R. M means DL2000 Marker. **(B)** Identification of the *CmMR1* knockout transformants via PCR amplification; For each strain, the knockout event was confirmed by PCR amplification using primer pairs KOG-F/R (for gene *CmMR1*, 1,381 bp), HYG-F/HY-R (for HYG, 740 bp), KOU-F/HY-R (for upstream, 2,236 bp), and YG-F/KOD-R (for downstream, 2,499 bp), individually. **(C)** Identification of the complemented transformants via PCR by primer pairs KOG-F/R, HYG-F/HY-R and NEO-F/R (neomycin-resistance gene fragment, 582 bp). **(D)** Expression of *CmMR1* at 72 hpi in the knockout and complemented transformants via RT-PCR amplification. *Actin* was checked as a control with Actin-F/Actin-R. M means DL2000 Marker.

To probe if *CmMR1* was expressed in the wild-type and mutant strains, the total RNA was extracted from the mycelial mass collected from colonies growing for 48 to 96 h on PDA plates and RNA samples were examined with RT-PCR amplification. The results showed that *CmMR1* expressed all the stage from the hyphal growth (48 h) to the pycnidial maturation (96 h) stage in the wild-type strain ZS-1. However, the expression could not be detected at all stage tested in ZS-1TN24363, suggesting that *CmMR1* was disrupted by the T-DNA insertion in ZS-1TN24363 (Figure 2A).

### Target Knockout and Gene Complementation

To confirm that *CmMR1* is responsible for the change in colony morphology in ZS-1TN24363, both targeted gene knockout and gene complementation were conducted. The strategy for targeted knockout of *CmMR1* is shown in Supplementary Figure S3. Three knockout candidates were obtained and named ΔCmMR1-2, ΔCmMR1-22, and ΔCmMR1-23, respectively. PCR amplification with three primer pairs confirmed the targeted knockout event. In all candidates, a 1,381 bp fragment could not be amplified with the primer pair KOG-F/R, but could be amplified from the genomic DNA of the wild-type strain ZS-1; furthermore, the HYG fragment (740 bp), and fusion DNA fragments (2,236 bp for upstream and 2,499 bp for downstream) could be amplified from the genomic DNA of the candidates, but not from the wild-type strain ZS-1 (Figure 2B). The expression of *CmMR1* could not be detected at 72 hpi in the candidates (Figure 2D).

The full length gene was amplified from the genomic DNA of the wild-type strain ZS-1 and transformed into the targeted knockout mutants via *Agrobacterium*-mediated transformation. Strains growing on G418 and hygromycin-containing medium were considered as transformants. Most transformants could produce dark pycnidia, and three of them, CmMR1-2C1, CmMR1-23C7, and CmMR1-22C10, were randomly selected for PCR amplification. The results showed that a 1,381 bp DNA fragment could be successfully amplified from the transformants and strain ZS-1. Furthermore, genes for both hygromycin-resistance fragment (740 bp) and neomycin-resistance gene fragment (582 bp) could be amplified (Figure 2C) and the expression of *CmMR1* could be detected at 72 hpi in the transformants (Figure 2D).

### Colony Morphology of the Target Knockout Mutant of *CmMR1*

**FIGURE 3:**
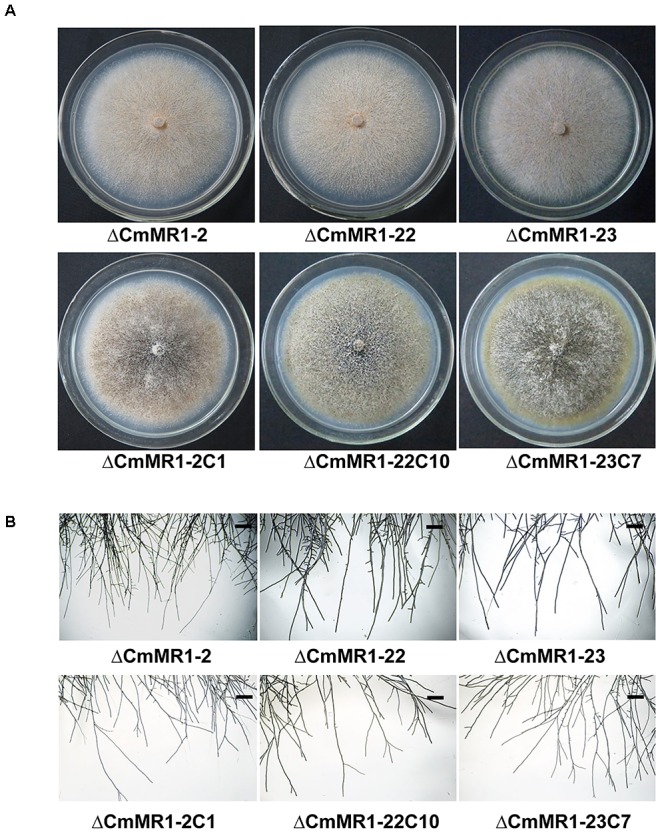
Comparison of colony morphology and hyphal tips of *CmMR1* targeted knockout mutants and complemented transformants. **(A)** Colony morphology. Images were taken 20 days after incubation on PDA at 20°C. **(B)** Hyphal tips of on PDA at 20°C for 4 days. Scale bar means 50 μm.

Compared to the wild-type strain, the targeted knockout mutants did not accumulate melanin and their colonies appeared whitish to orange in color. The mutants produced pycnidia and conidia similar to the wild-type strain; however, their pycnidia were not dark but white to orange in color (Figure 3A). The hyphal tips of the mutants were similar to those of the wild-type strain (Figure 3B). These characteristics were similar to that of the T-DNA insertional mutant (Figure 1B). The gene complemented transformants showed similar phenotypes as the wild-type strain ZS-1 (Figures 3A, 1B) and they could form dark pycnidia on medium. Thus, disruption of *CmMR1* was responsible for the phenotype of mutant ZS-1TN24363.

### Disruption of *CmMR1* Did Not Affect Hyphal Growth, Conidiation, and Parasitic Ability

**FIGURE 4:**
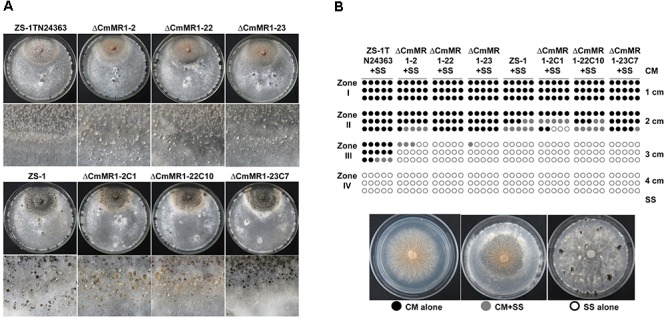
Parasitic activity assays of *C. minitans* to sclerotia of *S. sclerotiorum*. **(A)** A dual culture of *C. minitans* and *S. sclerotiorum* (PDA, 20°C, 20 days). **(B)** A schematic diagram showing the number of agar disks that gave rise to either *C. minitans*, *S. sclerotiorum* or both. Each circle represents a colony developed from a mycelial agar disk sampled from zones I, II, III or IV between the inoculation sites of *C. minitans* and *S. sclerotiorum* in a dual culture. Appearance of *S. sclerotiorum* colonies indicated that *C. minitans* could not parasitize *S. sclerotiorum* in this region. Similarly, the presence of *C. minitans* colonies suggested that *S. sclerotiorum* was eliminated by *C. minitans*. Occurrence of colonies of both *C. minitans* and *S. sclerotiorum* indicated that *C. minitans* was present at this region but could not destroy the hypha of *S. sclerotiorum.*

Compared to the wild-type strain ZS-1, the T-DNA insertional mutants, the targeted knockout mutants, and the gene complemented transformants differed negligibly regarding hyphal growth, mycelial mass, and conidiation (Table 2). When dual cultured with *S. sclerotiorum* on PDA plates, all the strains tested could parasitize the hyphae of *S. sclerotiorum*, with a slight stronger parasitic ability in the T-DNA insertional mutant (Figure 4). However, when inoculated on sclerotia of *S. sclerotiorum*, the parasitic ability did not differ significantly among all the mutants and the wild-type strain (Table 2). These results suggested that *CmMR1* had negligible effects on hyphal growth, conidiation, and parasitic ability.

**Table 2:** Comparison of growth rate, conidiation, biomass, and rot index.

Strains	Growth rate (mm/d)	Conidiation LOG (conidia/cm^2^)	Biomass (mg/plate)	Rot index
ZS-1TN24363	2.87 ± 0.04^A^	7.106 ± 0.054^A^	434.0 ± 25.72^AB^	72.17 ± 0.441^A^
ΔCmMR1-2	2.87 ± 0.05^A^	7.098 ± 0.092^A^	428.3 ± 40.00^AB^	66.58 ± 0.939^B^
ΔCmMR1-22	2.92 ± 0.05^A^	7.013 ± 0.030^A^	508.2 ± 17.60^AB^	69.67 ± 0.220^AB^
ΔCmMR1-23	3.04 ± 0.01^A^	7.048 ± 0.126^A^	398.0 ± 15.30^AB^	68.42 ± 1.460^AB^
ZS-1	2.99 ± 0.01^A^	6.923 ± 0.030^A^	374.3 ± 15.25^B^	72.08 ± 0.821^A^
CmMR1-2C1	2.97 ± 0.03^A^	6.926 ± 0.117^A^	390.3 ± 13.84^AB^	72.91 ± 0.961^A^
CmMR1-23C7	2.91 ± 0.04^A^	6.860 ± 0.059^A^	460.0 ± 29.96^AB^	69.08 ± 0.363^AB^
CmMR1-22C10	2.93 ± 0.01^A^	6.947 ± 0.118^A^	527.3 ± 20.63^A^	69.83 ± 0.712^AB^

*Different letters within a column indicate statistically significant differences (*P* < 0.01). Means and standard errors were calculated from three replicates*.

### Disruption of *CmMR1* Led to High Accumulation of ROS and Decreased Tolerance to UV Light

**FIGURE 5:**
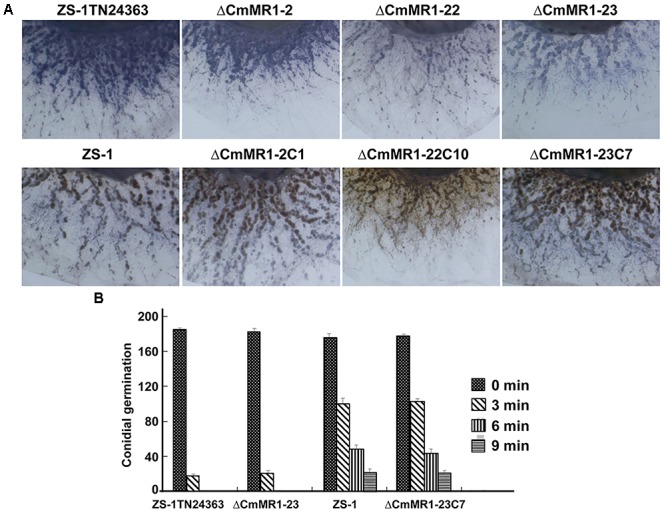
ROS production and tolerance to UV light of *CmMR1* disrupted mutants of *C. minitans.* **(A)** ROS production in strains was monitored by NBT staining (colonies were developed on PDA for 3 days under 20°C). **(B)** Conidial germination of all strains irradiated with UV light (20 J/cm^2^) for 0, 3, 6, and 9 min (20°C, 36 h).

The ROS content in *CmMR1* mutants was determined using NBT staining, and the results showed that T-DNA insertional mutant ZS-1TN24363 and the gene targeted knockout mutants had deeper color, whereas the wild-type strain ZS-1 and the gene complemented transformants were brown in color (Figure 5). This result suggested that the *CmMR1* disrupt mutants might accumulate higher levels of ROS. Without radiation treatment, the conidial germination of all mutants tested and the wild-type strain was similar at 36 h, and more than 90% conidia germinated. When treated with UV light for 3 min, 50% conidia of the wild-type strain and the gene complemented transformants did not germinate, while only 10% conidia of ZS-1TN24363 and ΔCmMR1-23 germinated. After treatment for 6 min, ZS-1TN24363 and ΔCmMR1-23 conidia could not germinate; however, 10%–25% of the strain ZS-1 and ΔCmMR1-23C7 conidia still germinated. A few of conidia of strain ZS-1 and ΔCmMR1-23C7 still germinated when treated for 9 min. Thus, results of ROS determination and UV treatment suggested that *CmMR1* might affect the UV and ROS tolerance of *C. minitans*.

### Expression of Melanin Synthesis Associated Gene *CmSCD*, *Cm3HNR*, and *Cm4HNR* Was Significantly Down-Regulated

**FIGURE 6:**
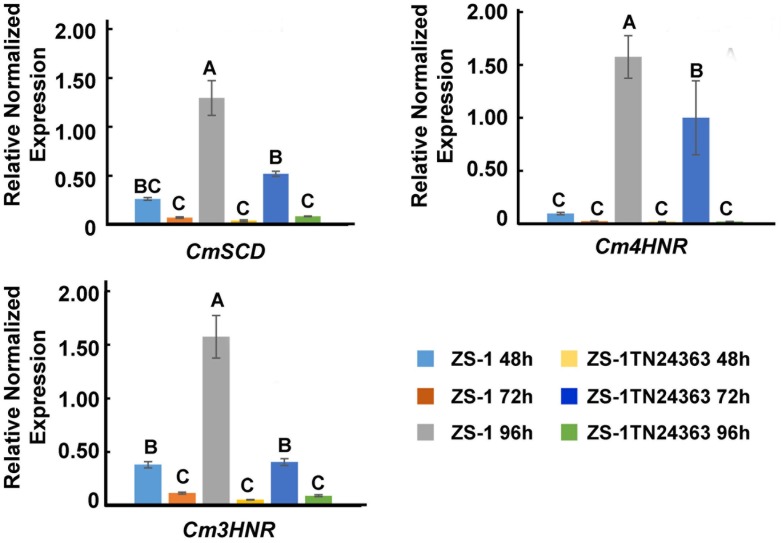
The expression of *CmSCD*, *Cm3HNR* and *Cm4HNR.* qRT-PCR amplification was used to check the expression of the genes from 48 to 96 hpi in strain ZS-1 and in mutant ZS-1TN24363. The *Actin* was used as an internal control to normalize the expression data with Actin-F/Actin-R. Significant tests were conducted with ANOVA and multiple comparing (significance level alpha = 0.01).

The expression of melanin synthesis associated genes was monitored by qRT-PCR. *CmSCD*, *Cm3HNR* and *Cm4HNR* were significantly down-regulated at the conidial production stage (96 hpi) in the mutant ZS-1TN24363 compared to those in strain ZS-1 (Figure 6), while expression of *CmLAC1* could not be detected either in strain ZS-1 or in mutant ZS-1TN24363.

## Discussion

*Coniothyrium minitans* is a well-known biological control agent. Previous studies have mainly focused on its growth, conidiation, and parasitical mechanism; however, studies regarding the survival property of this fungus are limited. *C. minitans* produces abundant melanin, which accumulates on the pycnidia and conidia; however, the melanin synthesis pathway and its functions are not known. In this study, we screened a T-DNA insertional melanin-deficient mutant ZS-1TN24363 and identified the transcription factor-encoding gene *CmMR1*. MR1 is a conserved fungal transcription factor, and in other fungi, this protein was also found to be associated with melanin synthesis. Here, we observed that the expression of *CmSCD*, *Cm3HNR* and *Cm4HNR* were significantly down-regulated at the conidial production stage (96 hpi) in mutant ZS-1T24363, suggesting that these genes were regulated by CmMR1. Previously, we demonstrated that disruption of a gene encoding PKS of *C. minitans* exhibited similar phenotypes as the *CmMR1* target knockout mutants (Xiang et al., unpublished), suggesting that the PKS pathway is involved in melanin synthesis.

Melanin is involved in the pathogenicity of pathogenic fungi (Henson et al., 1999). Melanin accumulates in the appressorium of *M. griesa*, and a melanin layer was present around the plasma membrane of the appressorium, which was essential for its pathogenicity (Chumley and Valent, 1990; Soanes et al., 2012). The melanin layers were also found in the appressorium of *C. lagenarium.* Melanin is also essential for the pathogenicity of human pathogenic fungi as melanin deficiency leads to reduction or complete loss in pathogenicity (Langfelder et al., 2003). Moreover, in *A. brassicicola*, the transcription factor AMR1 induces melanin biosynthesis and increases fungal virulence, and was speculated to be important for both competitive saprophytic and plant parasitic activities (Cho et al., 2012). In our study, *CmMR1* did not significantly affect the ability of *C. minitans* to parasitize *S. sclerotiorum*. This is possibly because *C. minitans* does not form an appressorium for penetrating host hypha (Huang and Kokko, 1988). The functions of the fungal transcription factor CMR1 may be slightly different; for example, in addition to the control of melanization, the *A. alternata* CmrA was reported to control spore development. The hyphal diameter and spore morphology changed and the production of conidia was significantly reduced when *CmrA* was knocked out (Fetzner et al., 2014). Fungi require melanin to tolerate environmental stress, such as UV irradiation and presence of oxidants. UV light induces the expression of *CMR1*, *PIG1*, and *BMR1* in *C. lagenarium*, *M. grisea*, and *B. oryzae*, and their hypha were melanized (Tsuji et al., 2000; Kihara et al., 2008). Recently, the transcription factor homolog of *BMR1* was identified in the basidiomycetous fungus *Grifola frondosa*, and it was induced by near-ultraviolet light and blue light (Kurahashi et al., 2015). In our study, we found that the conidia of *CmMR1* mutants were highly sensitive to UV light irradiation. *C. minitans* parasitized the sclerotia of *S. sclerotiorum* under the soil. Since *C. minitans* grows under similar temperature conditions to *S. sclerotiorum*, it could also grow on the lesions induced by *S. sclerotiorum* on the aerial parts of plants where it may be irradiated by UV light. Further, application of conidia on the aerial parts of crops is an important way of successfully controlling Sclerotinia diseases in the field, and enhancing conidial melaninization is a potential way of improving the on-field survival ability of crops.

## Conclusion

In this research, *CmMR1*, encoding a transcription factor MR, was cloned and the functions were studied in *C. minitans*, a mycoparasite of *S. sclerotiorum*. Knockout of *CmMR1* led to the shortage of melanin during conidiation and decrease of tolerance to UV light. Our result may enhance the understanding of melanin in the ecology of *C. minitans* on molecular level.

## Author Contributions

CL, DJ, and YF designed the research and wrote the paper. CL, XY, CQ, HZ, and JC executed the experiments. JC, TC, JX, and YF performed the data and bioinformatics analyses. All authors read and approved the final manuscript.

## Conflict of Interest Statement

The authors declare that the research was conducted in the absence of any commercial or financial relationships that could be construed as a potential conflict of interest.

## Supplementary Material

FIGURE S1 *CmMR1* disrupted in the T-DNA insertional mutant ZS-1TN24363 of *C. minitans.* **(A)** The result of hiTAIL-PCR. Arrow shows the target product of the secondary PCR. **(B)** The sketch of the flanking genomic DNA segment and the T-DNA border. **(C)** T-DNA insertional event in mutant ZS-1TN24363. **(D)** Analysis of putative conserved domains coded by *CmMR1*.

FIGURE S2 Phylogenetic analysis of CmMR1 and proteins of other fungi. The alignment was performed with Neighbor-joining method of MEGA 6.0 program. Bootstrap values (%) obtained with 1000 replicates are indicated on branches and branch lengths correspond to genetic distance; A scale bar at the lower left corresponds to a genetic distance of 0.1.

FIGURE S3 The strategy of knockout with split-marker recombination.
